# First malaria in pregnancy followed in Philippine real-world setting: proof-of-concept of probabilistic record linkage between disease surveillance and hospital administrative data

**DOI:** 10.1186/s41182-024-00583-7

**Published:** 2024-02-08

**Authors:** Takuya Kinoshita, Fe Espino, Raymart Bunagan, Dodge Lim, Chona Daga, Sabrina Parungao, Aileen Balderian, Katherine Micu, Rutchel Laborera, Ramon Basilio, Marianette Inobaya, Mario Baquilod, Melecio Dy, Hitoshi Chiba, Takehiro Matsumoto, Takeo Nakayama, Kiyoshi Kita, Kenji Hirayama

**Affiliations:** 1 Department of Health Informatics, Graduate School of Biomedical Sciences, Nagasaki University, Nagasaki, Japan; 2 Department of Parasitology, Research Institute for Tropical Medicine, Department of Health, Muntinlupa City, Philippines; 3 National Tuberculosis Reference Laboratory, Research Institute for Tropical Medicine, Department of Health, Muntinlupa City, Philippines; 4 Department of Epidemiology and Biostatistics, Research Institute for Tropical Medicine, Department of Health, Muntinlupa City, Philippines; 5 Kilusang Ligtas Malaria, Provincial Health Office, Puerto Princesa City, Palawan Philippines; 6 Rural Health Unit, Punta Baja, Rizal, Palawan Philippines; 7 Center for Health Development MIMAROPA, Quezon City, Philippines; 8 Ospital Ng Palawan, Puerto Princesa City, Palawan Philippines; 9 School of Tropical Medicine and Global Health, Nagasaki University, Nagasaki, Japan; 10 Department of Health Informatics, Graduate School of Medicine and Public Health, Kyoto University, Kyoto, Japan; 11 Department of Host-Defense Biochemistry, Institute of Tropical Medicine, Nagasaki University, Nagasaki, Japan; 12 Department of Immunogenetics, Institute of Tropical Medicine (NEKKEN) and Interfaculty Initiative of Planetary Health, Nagasaki University, Nagasaki, Japan

**Keywords:** Malaria in pregnancy, Real-world data, Real-word evidence, Probabilistic record-linkage, Philippines

## Abstract

**Background:**

Although the Philippines targets malaria elimination by 2030, it remains to be a disease that causes considerable morbidity in provinces that report malaria. Pregnant women residing in endemic areas are a vulnerable population, because in addition to the risk of developing severe malaria, their pregnancy is not followed through, and the outcome of their pregnancy is unknown. This study determined the utility of real-world data integrated with disease surveillance data set as real-world evidence of pregnancy and delivery outcomes in areas endemic for malaria in the Philippines.

**Methods:**

For the period of 2015 to 2019, electronic data sets of malaria surveillance data and *Ospital ng Palawan* hospital admission log of pregnant women residing in the four selected barangays of Rizal, Palawan were merged using probabilistic linkage. The source data for record linkage were first and last names, birth date, and address as the mutual variable. The data used for characteristics of the pregnant women from the hospital data set were admission date, discharge date, admitting and final diagnosis and body weight on admission. From the malaria surveillance data these were date of consultation, and malaria parasite species. The Levenshtein distance formula was used for a fuzzy string-matching algorithm. Chi-square test, and Mann–Whitney *U* test were used to compare the means of the two data sets.

**Results:**

The prevalence of pregnant women admitted to the tertiary referral hospital, Ospital ng Palawan, was estimated to be 8.34/100 overall, and 11.64/100 from the four study barangays; that of malaria during pregnancy patients was 3.45/100 and 2.64/100, respectively. There was only one true-positive matched case from 238 women from the hospital and 54 women from the surveillance data sets. The overall Levenshstein score was 97.7; for non-matched cases, the mean overall score was 36.6 (35.6–37.7). The matched case was a minor who was hospitalized for severe malaria. The outcome of her pregnancy was detected from neither data set but from village-based records.

**Conclusions:**

This proof-of-concept study demonstrated that probabilistic record linkage could match real-world data in the Philippines with further validation required. The study underscored the need for more integrated and comprehensive database to monitor disease intervention impact on pregnancy and its outcome in the Philippines.

**Supplementary Information:**

The online version contains supplementary material available at 10.1186/s41182-024-00583-7.

## Introduction

Malaria, a life-threatening infectious disease caused by *Plasmodium* parasites, such as *Plasmodium falciparum* (*P. falciparum*) and *Plasmodium vivax* (*P. vivax),* continues to pose significant challenges to global health [1]. Despite considerable progress in prevention and control, malaria remains a major public health concern, particularly in regions with high transmission rates [2]. Among the populations most vulnerable to the disease are pregnant women, and an estimate of 120 million pregnant women are at risk of malaria (malaria in pregnancy or MiP). These patients can experience severe maternal and fetal outcomes, such as maternal anemia, low birth weight, and preterm delivery [3].

The Philippines targets malaria elimination by 2030 and implements a sub-national disease elimination approach to reach this goal [4]. The number of malaria cases has decreased over the years, from 19,102 cases in 2010 to 3150 in 2022 [5, 6]. Currently, only three provinces, Mindoro Occidental, Palawan, and Sultan Kudarat, report the disease. Over 95% of cases are in Palawan, where more than 60% are reported from Rizal municipality [7]. From 2005 until 2017, the Philippines utilized a paper-based malaria health information system called the Philippine Malaria Information System (PHILMIS) [8]. In 2018 it was replaced by an online malaria information system called OLMIS [9]. Malaria cases are diagnosed at the first point of contact in endemic communities and recorded in the malaria registry. The information is entered into a database in the rural health clinics each month and subsequently transmitted to a server that is accessible to the malaria coordinator of the provincial health office. The database is then forwarded to the National Malaria Control and Elimination Program [9].

The number of MiP cases in the Philippines has decreased from 45 cases in 2017 to 22 cases in 2021, and 26–66% were reported from Palawan [10]. Although the World Health Organization (WHO) recommends intermittent preventive treatment during pregnancy (IPTp), this intervention is not practiced in the Philippines [11]. In provinces that have been declared malaria-free, pregnant women who attend the prenatal checkups in barangay (village) health stations (BHS) and who report a fever are referred to the main rural clinic, where they are assessed for malaria along with other illnesses. However, even with the ongoing efforts of the established surveillance system in the Philippines [4], MiP patients are not followed through, and the outcome of their pregnancy is unknown.

In recent years, real-world evidence (RWE) has gained attention for conducting observational studies using administrative data, such as electronic health records and receipts of reimbursement claims data. Using such data enables better comprehension and generalizability regarding patient follow-up and traceability [12] unlike case–control and cohort studies which may sometimes not accurately reflect the real-world settings due to difficulties in patient comprehension, lost to follow-up, and selection bias [13, 14]. Utilizing such data allows retrospective monitoring of the course of pregnancy and delivery outcomes. among MiP patients. Administrative data from provincial emergency referral hospitals can play a critical role in managing high-risk pregnancies, including those that may result from malaria [15]. However, these hospitals are not accessible to high-risk groups in communities endemic for malaria. Most of the RWD studies reported are focused on imported malaria in European countries [16–18]. Only a few observational cohort studies regarding MiP patients have been reported using patient clinical data [19, 20].

Integrating the existing malaria surveillance data and administratively collected hospital RWD have the potential to provide valuable information to assess healthcare utilization and outcomes in the Philippines. However, these data sets are collected and analyzed separately. Furthermore, patient data are still collected and stored as handwritten paper charts and registries in many of the rural healthcare facilities in the Philippines and the quality of data recording poor. Record linkage techniques have emerged as a promising approach to bridge disease surveillance and hospital RWD gaps by integrating data from multiple sources and providing a more comprehensive picture of patient care [21–23]. Although these techniques have been applied deterministically and probabilistically in some registries for malaria and newborns [24, 25], their efficacy in matching patients across malaria surveillance and hospital administrative data in the Philippine context has not been attempted. The objective of this proof-of-concept study was to investigate whether record linkage can be applied to match malaria surveillance and hospital administrative data within the Philippine context and to develop RWE of pregnancy and delivery outcomes among women residing in areas endemic for malaria.

## Materials and methods

### Study site

**Fig. 1:**
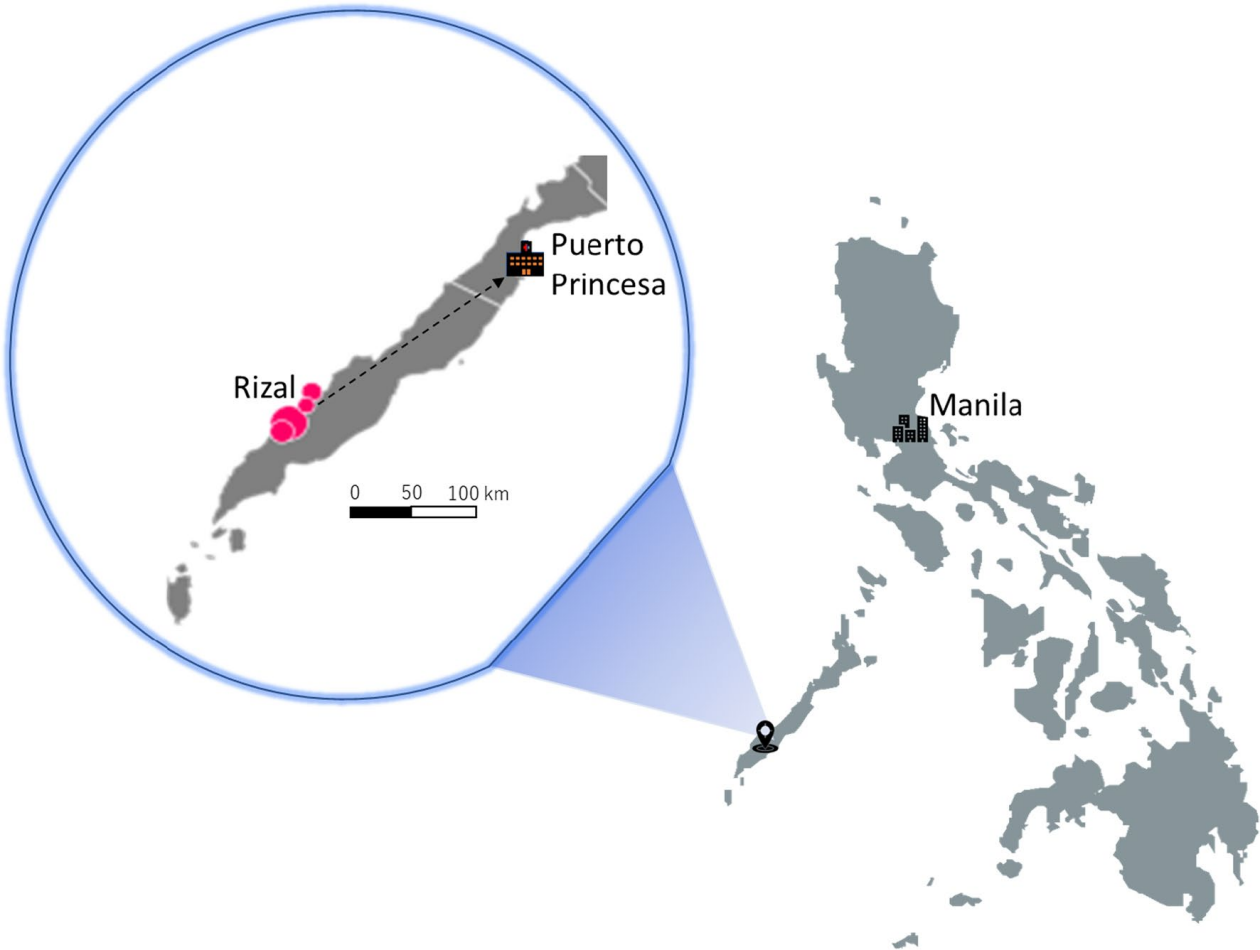
Study site and the distance to *Ospital ng Palawan*, the largest tertiary hospital in Palawan

Up to 88.8% of the malaria cases in the Philippines is due to *P. falciparum* followed by *P. vivax* (12.1%). From 2015 to 2019, 92.2–99.0% of malaria cases in the country, repectively, were reported from Palawan alone [7]. Palawan, thus, is a priority province for malaria elimination. In 2019, up to 62.0% of malaria cases were from the municipality of Rizal [26]. Rizal is a first-class municipality with a population of 56,162 and is 230 km south of Puerto Princesa City, the provincial capital of Palawan (Fig. 1). More than 80% of its land is timberland, 15% is agricultural, and the rest is built-up area. Subsistence farming, swidden agriculture, and fishing are the major sources of income for the residents. Almost 40% of the population is below the age of 15, the median age is 20 years, and the male to female ratio is 1.13 [27]. Rizal has 14 barangays (villages). Four, which we will call Barangay A, Barangay B, Barangay C, and Barangay D, where up to 47.2% of malaria cases were observed in 2016 [4], were selected for this study. Because reducing the number of malaria cases in Palawan is a high priority for the Department of Health, each barangay in Rizal has at least one health worker trained to diagnose malaria (by blood film microscopy or a rapid diagnostic test) and treat malaria at the point of contact. Individuals who consult for fever or who have a history of fever are tested for malaria. The *Ospital ng Palawan* (ONP) is the national referral hospital of the Philippines' Department of Health and is located more than 200 kms away in Puerto Princesa City.

### Source documents and data sets used

Maternal records from 2015 to 2019 archived in the Palawan provincial health office (PHO), BHS of barangays A, B, C, and D in Rizal, and Ospital ng Palawan (ONP) were reviewed. Table 1 lists these documents and the variables they contain. The Maternal and Child Health (MCH) program’s forms that were reviewed were the target client list for nutrition and the EPI program for under-fives (TCLNE) and the target client list for pregnant women (TCLP). The barangay health workers (also known as community health workers, or BHWs) are in charge of managing these forms within the BHS. In addition to the variables in Table 1, the TCLNE contains information that tracks the child’s development and immunization. Scanned copies of these were obtained after written permission was secured from the appropriate health authorities. From the PHO, we obtained the malaria electronic data sets for the period under study. Administrative records of all pregnant women between the ages of 15 and 49 who resided in Rizal and who were admitted to the Obstetric Department of ONP for any reason were also used. These documents were the hospital admission logbook, obstetrics and gynecology admission history form and clinical cover sheet. The paper-based target client lists were used to know the outcome of pregnancy for the MiP patients admitted to the ONP and who were admitted for reasons other than the delivery of their baby. The malaria surveillance data and hospital admission log are in electronic form and were the data sets used in the analysis. The source data used for record linkage were first name, last name, birth date, and address as a mutual variable. The following data were used for MiP and non-MiP pregnant patient characteristics: admission date, discharge date, admitting and final diagnosis from the hospital data, and the date of consultation, body weight on admission, and malaria parasite species in the surveillance data.

**Table 1:** Source documents and form details for data linkage of pregnant woman and follow through to birth outcome

Document			Information about mother												Information about baby	
Name	Paper/ Electronic)	LOCATION	Name			Date of birth	Age	Address^$^	Last menstrual period	Malaria diagnosis (date)	Pregnancy	Delivery			Pregnancy outcome	Birth weight/ length
			First	Middle or initial	Last							Date	Health facility	Type		
Target client list prenatal visits (TCLP)*	Line list/ Paper	*Barangay* health station	Not broken into parts			No	Yes	Yes	Yes	No	–	Yes	Yes	No	Yes	Yes
Target client list nutrition and EPI (TCLNE)*	Line list/ Paper	*Barangay* health station	Not broken into parts			No	No	No	–	–	–	Yes	No	No	Yes	Yes
Malaria surveillance data (PHILMIS)	Line list/ Electronic	Provincial Health Office	Yes	Yes	Yes	Yes	Yes	Yes	–	Yes	Yes	–	–	–	–	–
Hospital admission logbook^@^	Line list/ Electronic	*Ospital ng Palawan*	Yes	Yes	Yes	Yes	Yes	Yes	–	–	Yes	No	–	Yes	Yes	–
OB-GYN history form and clinical cover sheet^#^	Medical chart/ paper	*Ospital ng Palawan*	Yes	Yes	Yes	Yes	Yes	Yes	Yes	No	–	Yes	–	Yes	Yes	No

*PHILMIS* Philippine Malaria Information System, *OB-GYN* obstetrics and gynecology; *Forms used by the Maternal and Child (MCH) Health program, ^@^Includes full admission and discharge diagnosis, and pregnancy and maternal outcomes. ^#^ This is the name of the form. It is similar to a medical chart and includes the current and past pregnancy and/or gynecological history of the patient. The clinical cover sheet is a summary sheet. Matched case was given full treatment for malaria while confined in OnP. ^$^*Sitio* (hamlet) and/or *barangay* (village), – Not applicable

### Record linkage

**Fig. 2:**
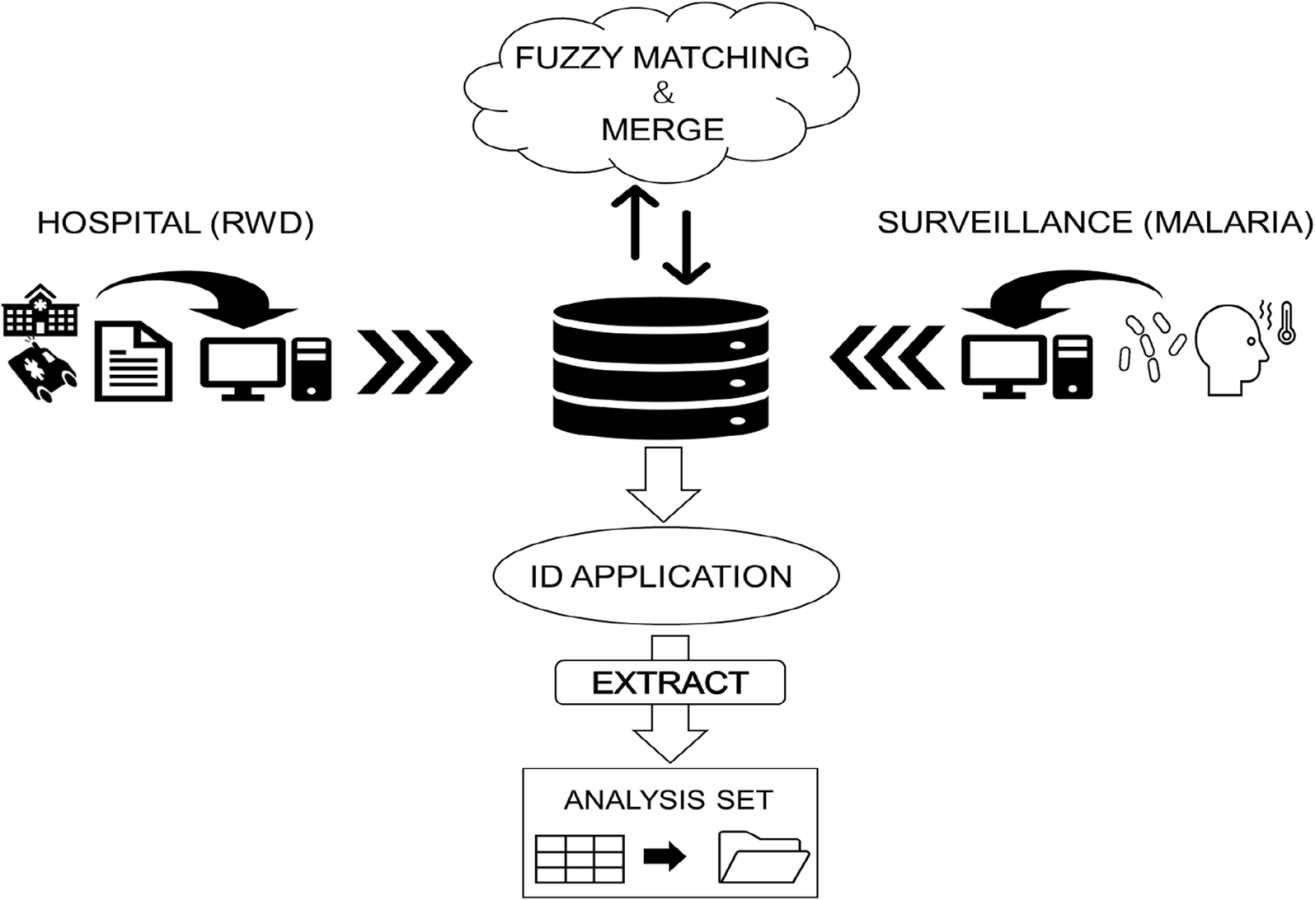
Concept of record linkage using fuzzy-matching for database development

Patient matching was explored using a probabilistic approach between hospital administrative and surveillance data. A fuzzy string-matching algorithm based on the Levenshtein distance formula (1) was employed [28]. After merging the two data sets using probabilistic record linkage, a unique nine-digit (two string and seven numeric: XX1234567) identification code was applied for further analysis. Patients with missing data such as all parts of the name (i.e., no entry for any of the name parts—first and last name), birthdate, or address were excluded (Fig. 2).

The principle of Levenshtein distance involves two string parameters and yields a numerical score that denotes the similarity between the two strings. This score is determined by calculating the Levenshtein distance (Lev_*a,b*_) between strings *a* and *b*, which represents the minimum number of insertions, deletions, or substitutions necessary to transform one string into the other. The lengths of strings *a* and *b* are denoted as *i* and *j*, respectively:

1 $${{\text{Lev}}}_{a,b}\left(i,j\right)= \{\left(i,j\right) \{{{\text{Lev}}}_{a,b}\left(i-1,j\right)+1 {{\text{Lev}}}_{a,b}\left(i,j-1\right)+1 {{\text{Lev}}}_{a,b}\left(i-1,j-1\right)+{1}_{\left({a}_{i}\ne {b}_{j}\right)} \,\, \mathrm{if }\left(i,j\right) =0, \,\,{\text{Otherwise}}.$$

**Fig. 3:**
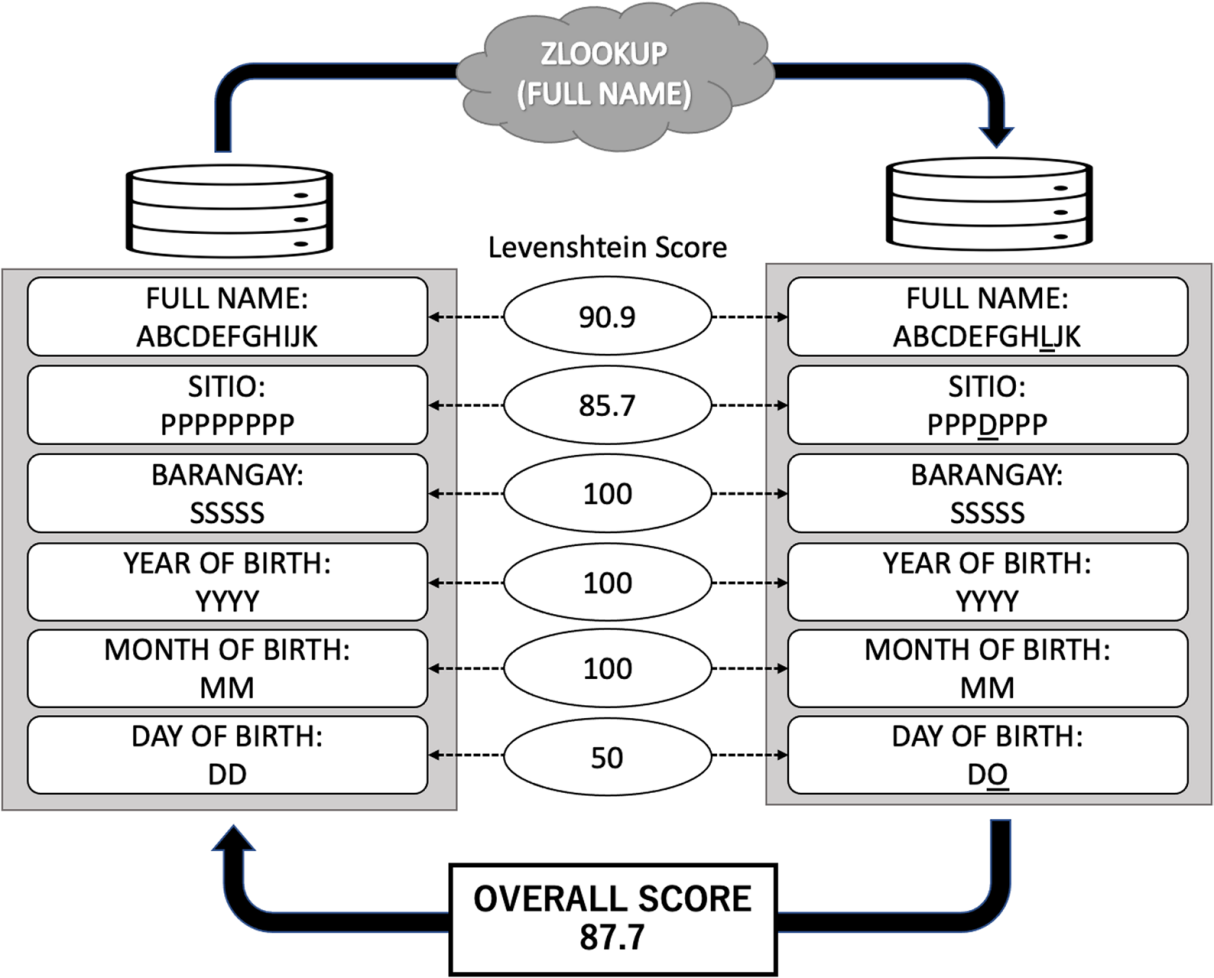
Example of the probabilistic record linkage at 70% threshold

The foundation of the score between the two strings is normalized between 0 and 100 by dividing the length of the longer string and subtracting the Levenshtein distance from 1 (2). To perform record linkage with fuzzy string matching, a threshold was implemented in necessitating a minimum similarity score for a match to be deemed valid. The highest score within the two strings across the data set was considered a match if it surpassed the threshold ranging from 0 to 100. This formula was used by applying a “Zlookup” function based on the following code provided for free on GitHub: < script src = “https://gist.github.com/andrei-m/982927.js”></script> This function searches for the nearest string from the two data sets, and scores can be obtained between them (Fig. 3). The JavaScript of the fuzzy matching was applied to the extension of Google Sheets for analysis.

2 $${\mathrm{Lev Score}}_{a, b}=\left(\frac{{1-{\text{lev}}}_{a,b} }{{\text{max}}\left(i,j\right)}\right)100>{\text{Threshold}}$$

### Main analysis

Probabilistic fuzzy matching was conducted by looking up aggregated strings of full names (last name and first name), followed by birthdate (year, month, and day), and address (barangay and sitio) from the hospital and surveillance databases. The score of each variable was calculated using the aforementioned formula, and the overall score was determined by dividing aggregated score with the number of variables. The score from the observed variables was plotted in three dimensions, and the frequency of the overall score was distributed within each patient. The proportion of true positives and false positives was calculated at thresholds ranging from 0 to 100 for both data sets. Three authors conducted the record linkage for validation and confirmation (TK, RB, and SE).

For the descriptive analysis, the background characteristics of the mutual variables used for the record linkage were compared between the non-matched patients from the hospital and surveillance. The mean of the patient’s full name string length and age were examined with 95% confidence intervals, respectively. In addition, the proportion of the four *barangays* and the registered year were investigated. For non-comparison variables, background characteristics obtained from hospital and surveillance data were described within matched and non-matched patients. From the hospital data, proportions of primigravida, miscarriage, multiple births, gestational weeks, readmission, comorbidities, and mean duration of hospitalization days were examined. From the surveillance data, these were the mean weight/kg, proportion of malaria parasite species (*P. falciparum*, *P. vivax*, or mixed), days until re-consultation, and weight change (kg).

### Statistical analysis

Data were analyzed in March 2023 and revised in April 2023. The Kolmogorov–Smirnov normality test was used to test if the null hypothesis of the matched scores from each patient distributed a normal distribution overall and for false positive patient scores. Then, the Shapiro–Wilk test was then applied for sensitivity analysis. Pearson’s Chi-square test was applied for comparing proportions, and Mann–Whitney’s *U* test was applied for comparing the means of the two individual data sets. A two-tailed test was applied to compare the two characteristics, with the criterion for statistical significance established at *α* = 0.05. *P* value less than 0.05 suggested that the observed difference between the characteristics was statistically significant. IBM SPSS version 28 was used for the analysis.

## Results

**Fig. 4:**
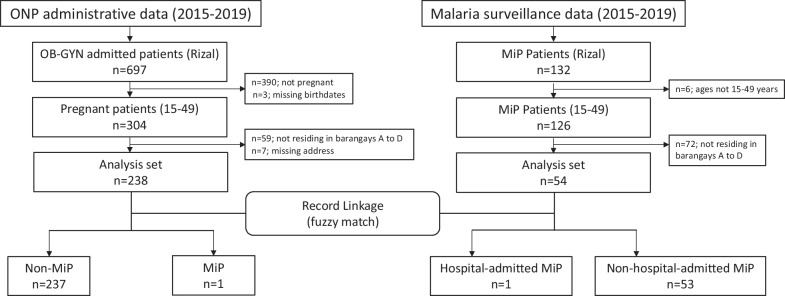
Flow diagram of patient selection and matching

**Fig. 5:**
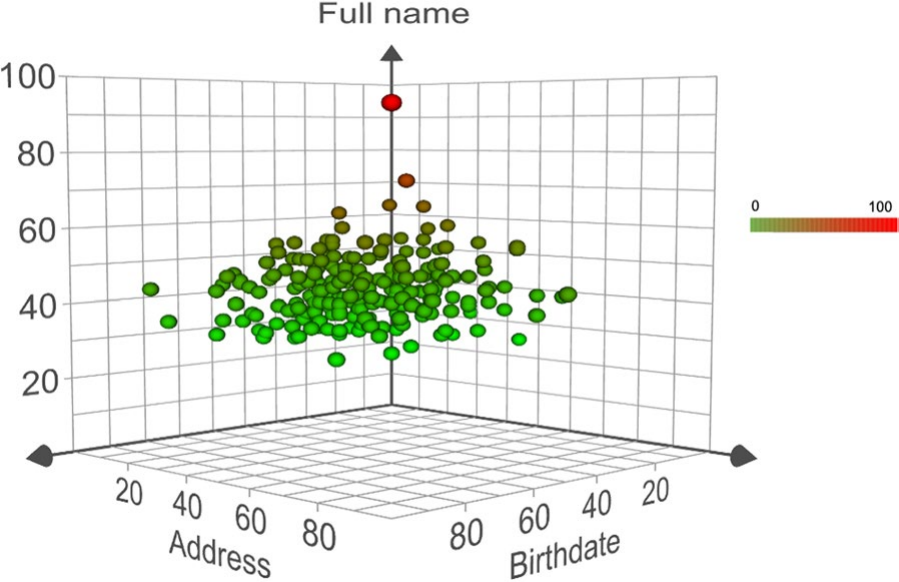
3D Plot of the Levenshtein Scores between hospital and surveillance patients

From 2015 to 2019, 697 women from Rizal were confined in ONP for various obstetric and gynecological conditions. Three-hundred and four women were pregnant and were between the ages of 15–49 years. During this same period, the malaria surveillance data set revealed that there were 132 women from Rizal diagnosed to have MiP and 126 were within the ages of 15–49 years. Two hundred thirty-eight (78.3%) of the pregnant women between the ages of 15–49 years admitted to ONP were from Rizal; 54 (42.9%) were from the four study barangays (Fig. 4). The prevalence of pregnant women admitted to ONP was estimated to be 8.34/100 overall and 11.64/100 from the four barangays. That of MiP patients was 3.45/100 and 2.64/100, respectively. There were 238 women from the hospital and 54 patients from the malaria surveillance on initial matching. There was one match that was true-positive with an overall score of 97.7 (Fig. 5).

According to the clinical characteristics obtained in the hospital, 31.5% of the pregnant patients were primigravida, and 1.9% had multiple births. The mean days of hospitalization and gestational weeks were 3.8 (3.4–4.1) days and 34.9 (33.5–36.2) weeks, respectively. Regarding pregnancy outcomes, 8.4% were miscarriages, and 5.3% were preterm deliveries. For comorbidities, 10.3% were pre-eclampsia and 5.3% were anemia (Table 2).

**Table 2:** Background characteristics from hospital data

	Non-MiP	MiP
	(*n* = 237)	(*n* = 1)
*Basic characteristics*		
Primigravida, *n*, (%)	80 (31.5)	No
Gestation weeks, mean, (95%CI)	34.9 (33.5–36.2)	25
*Labor outcome, n, (%)*		
Miscarriage	22 (8.4)	NA
Preterm labor	14 (5.3)	Not in labor
Twin	6 (1.9)	No
*Hospitalization outcome*		
Days of hospitalization, mean (95% CI)	3.8 (3.4–4.1)	18
Readmission, *n*, (%)	4 (1.2)	No
*Comorbidities, n, (%)*		
Pre-eclampsia	27 (10.3)	No
Anemia	7 (5.3)	√
Septic shock	0 (0.0)	√
Malaria	0 (0.0)	√

From the surveillance data, the mean weight of MiP patients was 43.9 kg (41.8–46.0) 90.5% of the parasites were *P. falciparum*, 5.6% were *P. vivax*, and 1.8% were mixed. There was also one MiP who had malaria twice and the second malaria episode was recorded 55 days after the first consultation (Table 3).

**Table 3:** Background characteristics from the surveillance data

	MiP	Hospital-admitted MiP
	(*n* = 53)	(*n* = 1)
Background characteristics		
Weight/kg, mean, (95% CI)	43.9 (41.8–46.0)	41
Parasite, *n*, (%)		
*P. falciparum*	48 (90.5)	√
* P. vivax*	3 (5.6)	No
Mixed infection	1 (1.8)	No
Re-consultation		
Cases *n*, (%)	1 (1.8)	√
Days after initial consultation	55	80
Weight change, kg, ±	0	− 4

Table 4 shows the comparison of the Levenshtein scores between the non-matched and matched MiP women. The specific scores of this matched case were 100 regarding address and birthdate and 91.6 for the full name with spelling discrepancies between the two data sets.

**Table 4:** Levenshtein score of the patients with fuzzy-matching

	Non-matched	Matched
	(*n* = 237)	(*n* = 1)
Total score, mean, (95% CI)	36.6 (35.6–37.7)	97.7
Full name	41.3 (40.3–42.3)	91.6
Birthdate (YYYYMMDD)	37.8 (35.4–40.1)	100
Year	44.6 (40.6–48.5)	100
Month	37.9 (34.3–41.5)	100
Day	22.3 (18.4–26.2)	100
Address (Aggregate)	31.0 29.1–32.9)	100
Barangay	47.0 (43.1–50.9)	100
Purok	16.5 (14.5–18.4)	100

Among the scores investigated in the non-matched patients, the mean overall score was 36.6 (35.6–37.7) and did not exceed more than 70 (Additional file 1: Fig. S1). When examining the normality of the matching scores, the distributions were not significant with the Kolmogorov–Smirnov normality test (*p* = 0.2) and were significant with the Shapiro–Wilk test (*p* =  < 0.001) overall. When excluding the true-positive matched patient, it was not significant under both the Kolmogorov–Smirnov normality test (*p* = 0.2) and the Shapiro–Wilk test (*p* = 0.344). There were no false-negative patients when validating the matched patients at thresholds from 0 to 90, but the only true-positive patient became false-negative at a threshold of 100. The proportion of true-positive patients surpassed the proportion of false-positive patients at the threshold of 70 (Additional file 1: Fig. S2).

When looking at the demographic difference of the mutual variables used for record linkage, the patient characteristics between the hospital data and surveillance data had a significant mean difference in the patient’s full name string length (13.5 letters and 12.2 letters) and age (27.7 years and 22.9 years), respectively (Table 5). In addition, there was a significant difference in the proportion of patients' residency at villages A (48.7% and 18.8%) and D (12.6% and 37.7%), respectively. When examining the cases regarding the year of diagnosis, there was a significant difference in 2017 (22.3% and 5.6%), 2018 (22.3% and 9.3%), and 2019 (11.5% and 35.2%), respectively.

**Table 5:** Difference of mutual background characteristics between hospital-admitted pregnant patients and MiP patients in the non-matched group

	Total	Non-MiP	MiP	*p *value
	(*n* = 290)	(*n* = 237)	(*n* = 53)	
*Basic characteristics, mean, (95%CI)*				
String length of full name	13.3 (13.0–13.6)	13.7 (13.3–14.1)	12.2 (11.6–12.8)	0.002
Age	26.9 (25.9–27.8)	27.3 (26.3–28.4)	22.9 (21.0–24.8)	0.0001
*Barangay (Address) n, (%)*				
Barangay A	137 (43.7)	119 (48.7)	10 (18.8)	< 0.00001
Barangay B	64 (20.4)	48 (20.3)	11 (20.7)	0.9
Barangay C	59 (18.8)	42 (18.0)	12 (22.6)	0.4
Barangay D	53 (16.9)	28 (12.6)	20 (37.7)	< 0.00001
*Registered Year n, (%)*				
2015	74 (23.6)	44 (23.5)	13 (24.5)	0.8
2016	79 (25.2)	54 (21.2)	14 (26.4)	0.3
2017	56 (17.9)	51 (20.4)	3 (5.6)	0.005
2018	61 (19.4)	55 (21.5)	5 (9.4)	0.03
2019	53 (16.9)	33 (13.5)	18 (33.9)	< 0.00001

### The matched case

The true positive matched case was of minor age and a resident of Barangay C. She was admitted to the ONP during her second trimester with the diagnosis of severe anemia of unknown etiology, was diagnosed with *P. falciparum* malaria and was confined for more than 2 weeks. She was registered in the malaria surveillance database twice. The first was on the day of her admission to ONP and the second was on her third trimester of pregnancy when she was diagnosed with falciparum malaria in the BHS. The TCLNE from the barangay was inspected afterwards, confirming the same first name (the mother's name) and last name (from the newborn’s last name) with the matched case recorded in the MiP. The matched case gave birth to a term baby girl, but the birth weight and height were missing. The name of the matched case was not listed in the TCLP, and, therefore, the place of birth (healthcare facility or home) is unknown. Thus, complete information on pregnancy and delivery outcomes was unknown.

## Discussion

This study is the first to our knowledge in the Philippines context to identify a pregnant woman who had severe malaria and was admitted to the largest provincial government referral hospital 230 km from her village. Documentation of severe malaria during her pregnancy would not have been matched deterministically nor followed through by the local health authorities of Rizal and Palawan, since both records had multiple errors in names. The case might have been counted as uncomplicated MiP with full-term delivery to a live baby girl at 38 weeks of gestation, while her hospital records reveal that the mother suffered severe malaria with anemia and septic shock during the second trimester. Missing such medical information as comorbidities could bias the results of retrospective studies, especially on pregnancy [29, 30].

In the current Philippine health system, malaria surveillance and hospital administrative data are assessed separately and are not adequately utilized or hybridized for research, such as epidemiology studies. On one hand, malaria surveillance data does not include detailed information about a woman’s pregnancy, such as the last menstrual period. On the other hand, hospital data lacks information about other endemic diseases in the community such as malaria unless these are suspected in a pregnant woman and diagnostic tests are performed. Information about the period of infection among women with MiP, as well as the duration of infection and treatment, is important for investigating the accurate relationship between the malaria infection and pregnancy outcome. Record linkage allows us to retrospectively estimate these conditions specifically first-trimester malaria infections, when most of these women could have been reported as non-pregnant at the first point-of-contact at the *barangay* health station. Resolving such concerns about the missing data of the last menstrual period that are often excluded in studies assessing anti-malaria treatment in pregnancy can possibly achieve a more robust result [19, 20, 24]. Therefore, integration and usage of RWD has the potential to provide information that allows a better assessment of the impact disease programs on vulnerable populations.

Despite national concerns and expectations for health prevention in a patient's life course approach there remain many gaps among multiple databases due to independent collection and inconsistency in recording. Thus, patient identification from multiple sources becomes difficult, and the utilization of data for assessing the disease becomes complicated. Malaria during pregnancy will be recorded in the malaria surveillance if the pregnant woman consults the rural clinic for fever or discloses her fever history during her prenatal check-ups. The matched case in this study was taken to ONP and admitted because of symptoms of severe anemia and septic shock. We later discovered from malaria surveillance records that she was also poorly nourished which could affect fetal growth. To fill in these gaps, universal health coverage and data harmonization such as applying unique patient identifiers with a uniformed method for health checkup registries, newborn registries, surveillance, and healthcare claims data, are important for better comprehension of the disease and for improving clinical outcomes. Thus, probabilistic record linkage could play such a role for underutilized data.

When looking closely at the background characteristics of the mutual variables used for matching, there was a significant demographic difference between pregnant women admitted to ONP and MiP patients with regard to string lengths of full name, age, and village of residence. These differences may have reflected the result of matching scores resulting in only one true-positive match. In addition, from the non-MiP patient’s characteristics at the hospital, about a third were primigravida; furthermore, 10% were diagnosed with preeclampsia. Although outcomes for newborns were not obtained for non-matched patients, the mean age between MiP and non-MiP patients is significantly different and needs to be adjusted when assessing maternal and newborn outcomes. With age as an example, propensity score matching (PSM) can be applied to observational studies to reduce biases when examining the impact of exposure or intervention. This is done through adjusting similar propensity scores, such as patient characteristics, co-morbidities, disease severity and treatment to ensure a balanced distribution of observed covariates between the two groups [31, 32]. However, studies using this method are limited to malaria and should be applied when using RWD for observing the associated risks [33]. Although we followed the course of pregnancy and delivery within the true-positive matched case, important variables such as drug treatment and duration could not be investigated unless patient charts were reviewed. These factors can be included in the analysis after integrating maternal clinical information from medical charts and by increasing the number health facilities in the review.

Considering the probabilistic record linkage, the proportion of true-positive patients exceeded when the 70% threshold was set for fuzzy matching in this study. In previous studies, 80% is said to be used for assuring true-positivity [12, 34]. This was probably affected due to the majority of false-positive patients which was around 36.7 compared to only one true-positive patient with a score of 97.7. Although we could not find any false negative matches in this study, false-positive patients could overlap in between the thresholds, and therefore, validation within the overlapping patient’s scores is necessary as more true-positive matches increase [35]. Alongside that, applying independent weights to missing data and prioritizing the variables depending on likelihood of matching can lead to a more robust result for reliability and accuracy of true-positive matches [22, 36]. Calculation of ROC curves and AUC scores are necessary when assessing the precision and recall of the fuzzy matching [37]. This study, however, contained only one true-positive match, and therefore, we could not investigate the aforementioned approach. Incorporating these aspects for validation are necessary in the future development of the database.

Though this record linkage is useful for utilizing multiple databases, there are still many obstacles to developing a comprehensive real-world database for epidemiological studies. We limited our search to patients admitted to the largest referral hospital over 200 km away from Rizal, a highly endemic malaria area and only one patient was considered a true-positive match. A majority of the MiP patients registered in the surveillance system remain unidentified; therefore, our results cannot be generalized. It is also possible that other women with MiP might have delivered their babies in health facilities closer to home.

## Conclusion

This proof-of-concept study demonstrated that using probabilistic record linkage could match various data obtained in the real-world setting of the Philippines which allowed us to investigate and follow the course of pregnancy and delivery among MiP patients. Integrating RWD with surveillance data to monitor events during the course of pregnancy revealed information which otherwise would remain unknown when surveillance databases alone are reviewed for assessing disease program impact. Although validation of the probabilistic record linkage is still needed to conduct future studies, enhancing these underutilized data may offer a possibility on impacting the maternal and newborn related to malaria in the Philippines.

### Supplementary Information

**Additional file 1: Figure S1.** Distribution of the Overall Levenshtein Scores. **Figure S2.** refers to the outcome of this procedure.

## Data Availability

Because data sets contain patient identifiers, it is not publicly available. The information could be made available upon review and approval by the RITM IRB of the requisitioner’s protocol which must have a written approval of the ethics committee of the requisitioner’s home institution.

## Abbreviations

AUC: Area under the ROC curve
BHS: Barangay health station
BHW: Barangay health worker
DOH: Department of Health
IPTp: Intermittent treatment of malaria during pregnancy
MCH: Maternal and Child Health
MiP: Malaria in pregnancy
OB-GYN: Obstetics and gynecology
OLMIS: Online malaria information system
ONP: Ospital ng Palawan
PHILMIS: Philippine Malaria Information System
PHO: Provincial Health Office
ROC: Receiver operating characteristic
RWD: Real world data
RWE: Real world evidence
TCLNE: Target client list for nutrition for the EPI Program
TCLP: Target client list for pregnant woman
WHO: World Health Organization
