# Supplementary material for: First malaria in pregnancy followed in Philippine real-world setting: proof-of-concept of probabilistic record linkage between disease surveillance and hospital administrative data
Source: Trop Med Health. 2024 Feb 8;52:17. doi: 10.1186/s41182-024-00583-7 (PMC10851569; doi:10.1186/s41182-024-00583-7)
Supplement: Supplementary file 1 — Additional file 1: Figure S1. Distribution of the Overall Levenshtein Scores. Figure S2. refers to the outcome of this procedure. [file 41182_2024_583_MOESM1_ESM.docx]

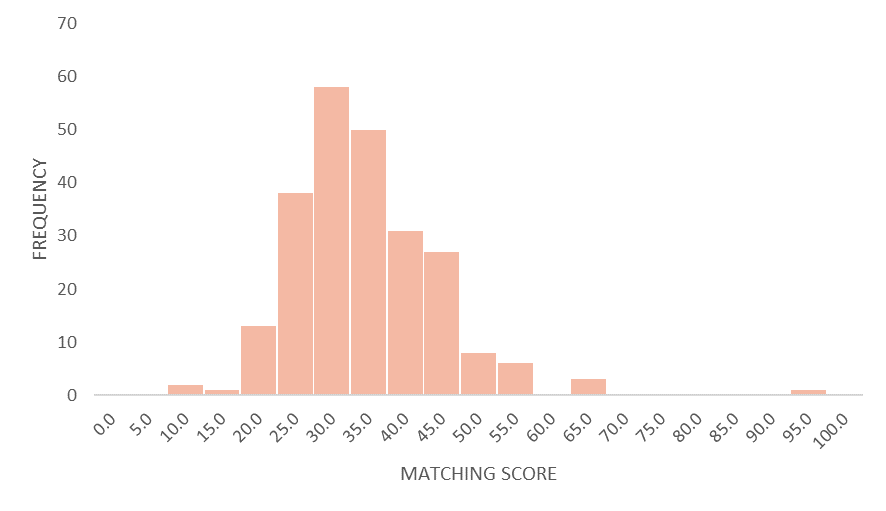


Figure S1. Distribution of the Overall Levenshtein Scores


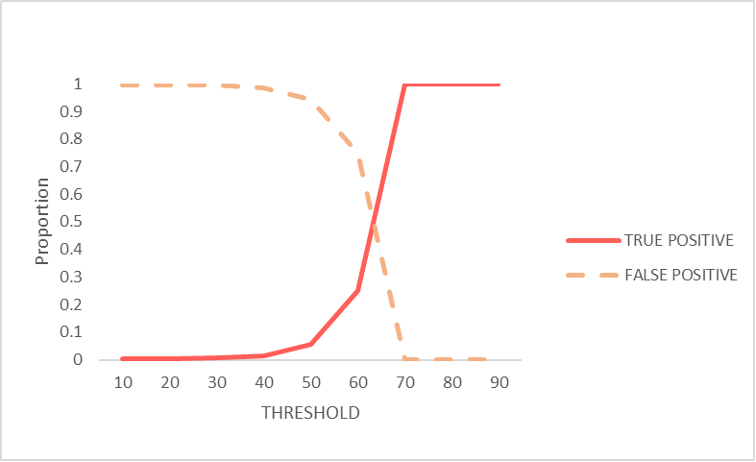


Figure S2. Proportion of True-Positive and False-Positive matches at each Threshold
